# The impact of physical exercise on college students’ interpersonal skills: the chain mediation effect of social anxiety and peer relationships

**DOI:** 10.3389/fpsyg.2026.1846593

**Published:** 2026-07-16

**Authors:** Yifeng Fu, Yong Jiang

**Affiliations:** School of Physical Education, Liaoning Normal University, Dalian, China

**Keywords:** chain mediation, interpersonal skills, peer relationships, physical exercise, social anxiety

## Abstract

**Introduction:**

Interpersonal skills are an important component of college students’ social adaptation and directly are associated with the quality of their social interactions and mental health. Physical exercise is an important means of supporting physical and mental health and is positively associated with the development of college students’ interpersonal skills. This study explores the association between physical exercise and college students’ interpersonal skills and the chain mediation effect of social anxiety and peer relationships.

**Methods:**

A questionnaire survey was conducted on 813 college students using the Physical Exercise Level Scale, interpersonal skills Scale, Social Anxiety Scale, and Peer Relationship Scale. Using Models in PROCESS Macros 6 and Bootstrap methods were employed to test and analyze the mediating effects of social anxiety and peer relationships.

**Results:**

① Physical exercise was significantly positively correlated with interpersonal skills, with a significant direct predictive effect; ② Physical exercise negatively predicted social anxiety, which in turn was negatively associated with peer relationships and interpersonal skills, while peer relationships positively predicted interpersonal skills; ③ Social anxiety and peer relationships played a statistically significant chain-mediated role between physical exercise and interpersonal skills, although the indirect effect sizes were relatively small. These formed three mediation pathways: Physical exercise → social anxiety → interpersonal skills (path 1), physical exercise → peer relationships → interpersonal skills (path 2), and physical exercise → social anxiety → peer relationships → interpersonal skills (path 3).

**Conclusion:**

Physical exercise is not only directly associated with better interpersonal skills among college students but also indirectly associated via sequential pathways involving reduced social anxiety and enhanced peer relationships.

## 1 Introduction

The World Health Organization (WHO) explicitly states in its Guidelines on physical exercise and Sedentary Behavior that adolescents should engage in at least 1 h of moderate to vigorous physical exercise daily (WHO, 2020) to promote physical and mental health and reduce the potential risks associated with sedentary behavior (Arug, 2016; Hallal et al., 2012; Strain et al., 2024). The Ministry of Education, in collaboration with 17 other departments, issued the “Special Action Plan for Comprehensively Strengthening and Improving Student Mental Health Work in the New Era (2023–2025),” which emphasizes the need to further prioritize student mental health work and particularly focus on the mental health status of college students. As the backbone of national development, college students’ physical and mental health and social skills are not only crucial for individual growth but also closely linked to the overall vitality of society. However, college students currently face widespread issues of insufficient physical exercise. For example, a large-sample study published in Frontiers in Public Health reported that 44.23% of Chinese college students are physically inactive (Zhang L. et al., 2025). A meta-analysis of 40 studies further indicated that the overall prevalence of physical inactivity among Chinese college students is 31.4% (95% CI: 23.1%, 39.7%) (Li and Zhao, 2024), which is associated with psychological and social adaptation challenges such as heightened social anxiety and strained peer relationships—issues that have become global educational concerns. Research indicates that regular physical exercise is associated not only with better physical fitness but also indirectly with social interaction abilities through mechanisms such as emotional regulation and enhanced self-efficacy (Elias, 2018). However, the specific pathways through which physical exercise is associated with interpersonal skills require further exploration.

Interpersonal skills are a core dimension of college students’ social adaptation abilities, manifested as an individual’s comprehensive ability to establish and maintain harmonious relationships in social contexts (Ye, 2019). They have significant impacts on an individual’s mental health, self-awareness development, alleviation of anxiety and depression, social adaptation abilities, formation of social responsibility, as well as academic adaptation and academic achievement (Huang et al., 2016; Liao et al., 2016). However, environmental changes and competitive pressures during the university stage often correlate with increased levels of social anxiety among students, manifested as excessive worry and avoidance of social situations, thereby hindering the healthy development of peer relationships (Zhang P. et al., 2025). Notably, physical exercise may serve as a potential correlate of better interpersonal skills through a dual pathway: lower social anxiety and stronger peer support. Physical exercise is associated not only with the prevention or delay of diseases but also with better mental health (Zhang et al., 2017). In the field of exercise psychology, the psychological benefits of physical exercise in interpersonal communication have garnered significant attention (Mao and Guo, 2013). Social anxiety, as an individual’s negative emotional response to social evaluation, may be related to the emotional release and self-confidence brought about by physical exercise (Clark, 2021); while peer relationships, as an important component of college students’ social support networks, may be strengthened through exercise forms such as team sports and collaborative activities (Smith et al., 2019). However, existing studies have primarily isolated the effects of physical exercise on single psychological variables, and systematic research on the sequential mediating path of the “social anxiety–peer relationship” chain remains lacking.

This study adopts a positive psychology framework, viewing physical exercise as a dynamic correlate of college students’ interpersonal skills, and focuses on analyzing the sequential mediating role of social anxiety and peer relationships in this association. From the perspective of positive psychology, physical exercise is viewed not merely as a behavioral activity but as a resource that fosters psychological strengths, including emotional regulation, self-efficacy, and social connectedness. Social anxiety can be conceptualized as a deficit in emotional regulation, while peer relationships represent a key source of social support. This framework guides our hypothesis that physical exercise may be sequentially associated with better interpersonal skills first through lower social anxiety, which in turn is associated with more positive peer relationships.

## 2 Theoretical foundation and assumptions

### 2.1 The association between physical exercise and college students’ interpersonal skills

Physical exercise is an activity that primarily involves physical training and exercise loads, and it is associated with enhanced physical fitness, better health, and the maintenance of a certain level of physical movement capacity through leisure, health preservation, and the cultivation of psychological intelligence (Xi, 2004). Physical exercise is not merely a means of physical training; during the exercise process, coordination and cooperation with peers, shared progress, attitudes toward setbacks and difficulties, teamwork abilities, and disciplinary awareness are all related to the mental health of exercisers, and are associated with higher levels of empathy, self-control, self-confidence, and self-esteem (Chen, 2023; Gong and Yang, 2023; Malinauskas and Malinauskiene, 2021; Qin et al., 2023). Interpersonal skills refer to the psychological characteristics that enable individuals to effectively engage in interpersonal communication activities and ensure their smooth progression during interactions with others (Xu, 2022). These skills are one of the key abilities for individuals to adapt to society, encompassing capabilities such as proactive communication, appropriate refusal, self-disclosure, conflict management, and emotional support (Jin et al., 2023). Interpersonal skills are not only crucial for the personal growth and development of college students but also play a positive role in social harmony and stability. Therefore, how to cultivate college students with good interpersonal skills has become a focal point in modern education. Research has found that physical exercise is positively correlated with interpersonal skills (Fu, 2022). Physical exercise is associated with a greater willingness to actively interact with others, and with more adaptive cognitive processes and communication skills (Cong et al., 2020; Shi et al., 2019; Yan et al., 2020). Physical exercise is also associated with positive physical and mental states, and such states are linked to more active and proactive social interactions, which in turn are associated with better interpersonal skills (Chen, 2024). Based on this, research hypothesis H1 is proposed: physical exercise positively predicts interpersonal skills.

### 2.2 The mediating effect of social anxiety

Social anxiety is a common psychological issue among adolescents, primarily manifested as persistent feelings of shyness, tension, fear, anxiety, and other negative emotional responses in certain or multiple social settings (Arditte Hall et al., 2019; Nelemans et al., 2019). Interpersonal skills, as the foundation of social survival, are the starting point and core form of human social interaction (Wang, 2020). In recent years, communication barriers have been associated with frequent campus issues, reflecting the inadequacy of contemporary college students’ interpersonal skills, making discussions on college students’ social interactions particularly necessary (Luo, 2015). A review of previous studies indicates that physical exercise is associated with social anxiety (Liu and Tan, 2024). The role of physical exercise in being linked to lower social anxiety and higher psychological resilience and self-efficacy is increasingly recognized (Li et al., 2024; Shang et al., 2023). Activities such as baseball and beach games have been found to be associated with fewer symptoms of social anxiety (Sun and Meng, 2017). Consistent physical exercise is associated with more positive social networks and support systems, as well as lower levels of social anxiety (Menescardi and Estevan, 2021), and these factors are in turn associated with better interpersonal skills. Regarding the relationship between social anxiety and interpersonal skills, scholars generally agree that there is a negative correlation between the two (Li and Han, 2016; Wang et al., 2013). Further research indicates that social anxiety is related to interpersonal skills, and lower levels of social anxiety are associated with greater peer acceptance and higher levels of interpersonal skills (Deng et al., 2013). When an individual’s level of social anxiety is lower, their performance in interpersonal interactions tends to be more confident and natural, which is associated with better interpersonal skills. Based on the above analysis, this study proposes hypothesis H2: Social anxiety mediates the relationship between physical exercise and interpersonal skills among college students.

### 2.3 The mediating effect of peer relationships

Peer relationships refer to interpersonal relationships characterized by shared activities and mutual collaboration among individuals of the same or similar age (Liu et al., 2022; Zhou and Zhou, 2021). Some studies have shown that physical exercise is associated with greater cooperation and support among college students, which in turn is associated with more positive peer relationships (Qin and Qin, 2023). Cooperation and interaction in team sports are associated with stronger trust and reliance among peers, and with fewer instances of peer rejection and exclusion. Additionally, physical exercise is associated with better physical fitness and self-confidence, which are also linked to more positive peer relationships and better interpersonal skills. Positive and negative peer relationships have significantly different associations with individuals’ positive emotions and interpersonal skills. Positive peer relationships are associated with lower stress and fewer negative emotions such as loneliness, depression, and anxiety in college students’ academic and daily lives, and are associated with better interpersonal skills. Negative peer relationships, however, are associated with lower academic performance (Yan et al., 2021) and with higher risk of internalizing problems such as depression (Yan and Li, 2021), anxiety (Zhong et al., 2017), and related issues (Han et al., 2020). One study found that peer relationships are positively correlated with interpersonal skills. Students who establish safe and stable relationships with peers tend to interact better with others, strengthen interpersonal relationships, and feel more relaxed in subsequent social activities (Tang, 2021). These studies suggest that physical exercise may be associated with the development of positive peer relationships, and that positive peer relationships are associated with higher self-esteem and self-efficacy, which in turn are associated with better interpersonal skills (Zhou et al., 2024). Based on the above analysis, this study proposes hypothesis H3: Peer relationships mediate the relationship between physical exercise and college students’ interpersonal skills.

### 2.4 The chain mediation effect of social anxiety and peer relationships

Social anxiety was first described by Gelder and Mark in the UK in 1966, referring to individuals who avoid public activities due to fear of social situations, such as speaking in public (Marks and Gelder, 1966). Peer relationships are harmonious, equal interpersonal relationships established through communication and interaction between peers or individuals of similar age (Zhou, 2024). Previous research has shown that higher social anxiety is associated with poorer peer relationships (Wang et al., 2020). Students with social anxiety often experience more peer harm, lower peer acceptance, and higher levels of loneliness (Biggs et al., 2012). Students with severe social anxiety may encounter difficulties in interacting with peers, which may be associated with a vicious cycle of emotional distress and social anxiety. Additionally, social anxiety is negatively correlated with peer relationships (Shen, n.d.). The chain mediating effect of social anxiety and peer relationships can be understood from an integrated perspective of social cognitive theory and self-determination theory (Bandura, 1985; Deci and Ryan, 1985). From a social cognitive standpoint, physical exercise provides a context for mastery experiences and vicarious learning, which enhance self-efficacy beliefs and reduce social evaluative fears—key components of social anxiety. Reduced social anxiety, in turn, enables individuals to approach social situations with greater confidence and less avoidance, thereby facilitating the formation and maintenance of positive peer relationships. This sequential logic is also consistent with the process model of emotion regulation (Gross, 1998), in which successful antecedent-focused regulation (e.g., reducing anxiety through exercise) subsequently improves the quality of social interactions (i.e., peer relationships), which then feeds back into broader social competence (i.e., interpersonal skills). Moreover, the proposed temporal sequence is grounded in developmental and social psychological perspectives: changes in internal emotional states (social anxiety) are conceptually prior to changes in external relational outcomes (peer relationships), as the quality of peer interactions is typically contingent upon the individual’s emotional availability and social approach tendencies. Based on the above analysis, this study proposes hypothesis H4: Social anxiety and peer relationships play a chain mediating role in the association between physical exercise and college students’ interpersonal skills. We note, however, that the cross-sectional nature of our data does not allow for empirical testing of temporal precedence; this limitation is addressed in section 6.

**FIGURE 1:**
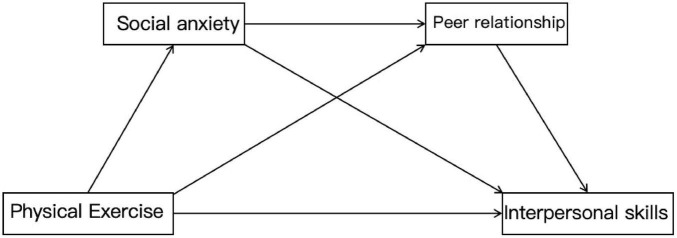
Hypothesis model of the impact of physical exercise on interpersonal skills.

Based on this, the research hypothesis model is constructed as shown in Figure 1.

## 3 Research subjects and methods

### 3.1 Research subjects

The research subjects of this study are the effects of physical exercise on college students’ interpersonal skills, as well as the mediating roles of social anxiety and peer relationships. During the research process, measurement times were separated and procedures were controlled to minimize the subjects’ speculation about the purpose of the measurement. To reduce the interference of common method bias, minimize the memory effect of the subjects, and avoid systematic bias caused by memory associations, the questionnaires in this study were administered in two separate sessions, with a 2-week interval between them. To match participants across the two rounds of the survey, each participant was assigned a unique anonymous code (composed of their date of birth), which was used in both data collection rounds. The matching process was completed using these codes prior to data analysis. The survey was conducted in June 2025 across multiple universities, disciplines, and majors in Sichuan Province, Chongqing Municipality, Henan Province, and Liaoning Province.

This survey was conducted online using the “Wenjuanxing” platform.^1^ A stratified random sampling method was employed. The target population was first divided into strata based on three variables: gender (male, female), grade (freshman, sophomore, junior, senior), and major (arts, sciences, physical education/arts). Within each stratum, a simple random sample of participants was drawn using a random number table. This procedure was designed to ensure proportional representation of each subgroup in the final sample. Links to the online questionnaire were distributed to participants via class WeChat groups and through course assistants. Participants were informed that their responses would be treated anonymously and used solely for research purposes. To ensure data quality, the following procedures were implemented: (a) All questions were marked as required to prevent omissions; (b) attention-check questions were embedded (e.g., “For this question, please select “Agree”)” to identify careless or inattentive responses; (c) the “One submission per device” feature was enabled in Wenjixing to prevent duplicate submissions; (d) completion times were monitored, and responses completed in under 3 min (one-third of the median completion time of 9 min) were excluded; (e) Screen for and exclude responses with linear patterns (selecting the same option on more than 80% of the Likert scale questions). After applying these criteria, 32 of the 845 questionnaires were excluded, resulting in a final valid sample of 813 (a response rate of 96.21%). The demographics of the participants are detailed in the Supplementary Table 1.

### 3.2 Research tools

#### 3.2.1 Physical exercise rating scale (PARS-3)

The physical exercise Rating Scale (PARS-3) revised by Liang (1994) was used. This scale aims to assess participants’ physical exercise intensity, duration, and frequency over the past month. Each indicator is scored using a 5-point Likert scale. physical exercise levels are measured across three dimensions: intensity, duration, and frequency. The total score is calculated as follows: physical exercise score = intensity score × (duration score – 1) × frequency score. Higher scores indicate greater levels of physical exercise participation. The PARS-3 is a composite index rather than a reflective scale, so traditional internal consistency (Cronbach’s α) is not the most appropriate indicator. Instead, its validity has been supported by prior research. In this study, the α coefficient was 0.896, reported only for descriptive purposes. However, it should be noted that the PARS-3 primarily reflects exercise volume (i.e., intensity, duration, and frequency) as a composite index, without distinguishing between different types of exercise (e.g., team-based versus individual sports) or the social context in which the exercise is performed (e.g., organized group activities versus solitary training).

#### 3.2.2 Interpersonal skills scale

This study adopted the Interpersonal Skills Scale developed by Buhrmester et al. (1988), which consists of 40 items and includes five dimensions: positive social interaction, appropriate response, conflict resolution, self-expression, and emotional support. A 5-point scoring method was used, with higher scores indicating higher interpersonal skills. The Cronbach’s α coefficients for the five dimensions are: 0.892, 0.890, 0.898, 0.897, and 0.895. In this study, the internal consistency reliability of the scale, as measured by Cronbach’s α coefficient, was 0.977.

#### 3.2.3 Social anxiety scale

This study used the Social Interaction Anxiety Scale developed by Peng et al. (2004) to assess individuals’ levels of social anxiety. The scale consists of 15 items scored on a 5-point scale, with items 3, 6, 10, and 15 scored in reverse and the remaining items scored in the forward direction. All item scores are summed, with higher scores indicating higher levels of social anxiety. In this study, the Cronbach’s α coefficient for this scale was 0.967, indicating that the scale reliably and consistently reflects participants’ levels of social anxiety.

#### 3.2.4 Peer relationship scale

The Peer Relationship Scale developed by Zou et al. (1998) was used. This scale consists of 30 items, divided into two dimensions: peer acceptance and peer fear and inferiority. The peer acceptance dimension includes items 1–20, with a Cronbach’s α coefficient of 0.974, while the peer fear and inferiority dimension includes items 21–30, the Cronbach’s α coefficient of 0.949. Among these, items 1, 3, 4, 7, 11, 17, and 21–30 are positively scored, while the remaining items (2, 5, 6, 8, 9, 10, 12, 13, 14, 15, 16, 18, 19, and 20) are negatively scored. A higher final score indicates better peer relationships. The Cronbach’s α coefficient for this scale is 0.982.

#### 3.2.5 Model fit indices for confirmatory factor analysis

In this study, AMOS 28.0 software was used to conduct confirmatory factor analysis on the measurement model to assess its overall fit. The results showed: χ^2^/df = 1.775, CFI = 0.952, TLI = 0.951, RMSEA = 0.031, and RMR = 0.045 (Table 1). All fit indices met or exceeded the acceptance criteria (χ^2^/df < 3, CFI > 0.90, TLI > 0.90, RMSEA < 0.08, RMR < 0.08), indicating that the measurement model fits the data well and possesses good construct validity.

**TABLE 1:** Model fit indices for confirmatory factor analysis.

Model	χ^2^/df	CFI	TLI	RMSEA	RMR
Criterion	<3	>0.90	>0.90	<0.08	<0.08
Four-factor model	1.775	0.952	0.951	0.031	0.045

### 3.3 Statistical methods

This study utilized SPSS 29.0 statistical software for data analysis. This study was not pre-registered. First, internal consistency reliability was assessed using Cronbach’s α coefficients for all multi-item scales, with separate α values reported for each subdimension where applicable.

Testing of statistical assumptions. Before conducting parametric tests (independent-samples *t*-tests, one-way ANOVA, and regression analyses), we examined the underlying assumptions of normality and homogeneity of variance. Normality of the distribution for each core variable (physical exercise, interpersonal skills, social anxiety, and peer relationships) was assessed using the Kolmogorov–Smirnov test (with Lilliefors correction) and visual inspection of Q–Q plots. Given that the Kolmogorov–Smirnov test can be overly sensitive in large samples (*N* = 813), we also examined skewness and kurtosis values; all were within the acceptable range of ∣2∣ for skewness and ∣7∣for kurtosis (West et al., 1995), indicating approximate normality. Homogeneity of variance for group comparisons (gender and grade) was tested using Levene’s test. The results showed that the assumption of homogeneity of variance was met for most variables (Levene’s test *p* > 0.05), except where noted. For regression analyses, residual plots were inspected and no severe violations were detected.

Descriptive statistics were computed to characterize the sample. Pearson’s correlation coefficients were calculated to examine bivariate relationships among physical exercise, interpersonal skills, social anxiety, and peer relationships.

To test the mediating roles of social anxiety and peer relationships, we used Model 6 of the PROCESS macro for SPSS, which estimates sequential mediation models. The significance of indirect effects was assessed using bootstrapping with 5,000 resamples to generate 95% bias-corrected confidence intervals (CIs). An indirect effect was considered statistically significant if the 95% CI did not include zero. In all regression analyses, gender and grade were included as control variables to account for their potential confounding effects.

The standardized beta coefficient *(β)* is reported as the measure of effect size in regression models, and *R*^2^ indicates the proportion of variance explained in the dependent variable.

#### 3.3.1 Rationale for group comparisons

Before testing the mediation hypotheses, we examined gender and grade differences in the core variables (physical exercise, social anxiety, peer relationships, and interpersonal skills). This preliminary analysis served two purposes. First, previous studies have consistently reported that male college students engage in more physical exercise than females and that social anxiety levels tend to be higher among females (Li et al., 2024; Shang et al., 2023). Likewise, grade differences in physical activity and social adaptation have been documented, with upper-year students facing distinct developmental transitions that may affect their exercise behavior and interpersonal functioning. Second, establishing whether significant group differences exist in these variables helps to determine whether demographic characteristics should be controlled for as covariates in the subsequent mediation model. If gender or grade significantly correlates with the mediators or outcome, including them as control variables can reduce confounding and improve the accuracy of the estimated mediation effects. Thus, these group comparisons are not an independent research goal but rather an essential preliminary step that informs the main chain mediation analysis.

## 4 Results and analysis

### 4.1 Common method bias test

Due to factors such as the measurement environment, questionnaire instructions, and context, questionnaire surveys may be subject to common method bias (Zhou and Long, 2004). A one-factor Harman test was conducted on the measurement data. Unrotated principal component analysis was performed using SPSS 29.0 for physical exercise, interpersonal skills, social anxiety, and peer relationships. The results showed that there were five factors with eigenvalues > 1, with the first factor explaining 37.272% of the variance, which is below the critical value of 40%. These results suggest that severe common method bias is not a major concern in this study. Nevertheless, given that all data were collected through self-report questionnaires, the possibility of social desirability bias or other response tendencies cannot be completely ruled out, and this limitation should be borne in mind when interpreting the findings.

### 4.2 Descriptive statistics and correlations

#### 4.2.1 Demographics

Descriptive statistical analysis shows (Table 2) that there were 813 college students in the sample, including 488 males, accounting for 60.1%, and 325 females, accounting for 39.9%. This indicates that there were more male college students than female college students participating in the survey. In terms of grade distribution, the largest group was seniors, with 236 students, accounting for 29.0%, followed by freshmen with 208 students, accounting for 25.6%, sophomores with 194 students, accounting for 23.9%, and juniors with 175 students, accounting for 21.5%. In terms of majors, there were 249 liberal arts students, accounting for 30.6%, 411 science students, accounting for 50.6%, and 153 arts and physical education students, accounting for 18.8%.

**TABLE 2:** Demographic characteristics of participants.

Variable	Form	Quantities	Percentage
Gender	Male student	488	60.1%
	Female student	325	39.9%
Grade	First-year university student	208	25.6%
	Second-year university student	194	23.9%
	Third-year university student	175	21.5%
	Fourth-year university student	236	29.0%

#### 4.2.2 Independent samples *t*-test

An independent samples *t*-test was used to analyze the differences between male and female college students in terms of physical exercise, interpersonal skills, social anxiety, and peer relationships (Table 3). The results showed that males had higher scores than females in physical exercise, interpersonal skills, and peer relationships, while females had higher scores than males in social anxiety. Significant differences were observed between males and females in physical exercise, interpersonal skills, social anxiety, and peer relationships.

**TABLE 3:** Comparison of differences in variables among students of different genders.

Variables	Gender	*N*	*M ± SD*	*t*	*p*	Cohen’s *d*
Physical exercise	Male	488	39.073 ± 31.855	15.655	<0.001	1.017
	Female	325	11.187 ± 18.851			
Interpersonal skills	Male	488	3.290 ± 0.896	10.027	<0.001	0.718
	Female	325	2.653 ± 0.870			
Social anxiety	Male	488	2.760 ± 1.043	−10.555	<0.001	1.040
	Female	325	3.546 ± 1.035			
Peer relationships	Male	488	3.144 ± 1.035	8.930	<0.001	1.034
	Female	325	2.483 ± 1.030			

Cohen’s *d* is a standardized mean difference, where 0.20 indicates a small effect, 0.50 a moderate effect, and 0.80 a large effect. All effect sizes in this table are close to or exceed 0.80, indicating that the gender differences are of considerable practical significance.

#### 4.2.3 ANOVA one-way analysis

ANOVA One-way was used to analyze the differences between college students of different grades in terms of physical exercise, interpersonal skills, social anxiety, and peer relationships. The results showed (Table 4) that senior students differed significantly from students in the other three grades in terms of physical exercise, interpersonal skills, social anxiety, and peer relationships.

**TABLE 4:** Comparison of differences in various variables among students of different grades.

Relevant variable	Grade	*M ± SD*	*F*	*P*	*η^2^*	*Tukey HSD*
Physical exercise	1 (208)	26.134 ± 30.717	13.483	<0.001	0.048	a
	2 (194)	23.304 ± 27.565				a
	3 (175)	21.514 ± 27.214				a
	4 (236)	38.059 ± 32.874				b
Interpersonal skills	1 (208)	2.858 ± 0.922	33.830	<0.001	0.111	a
	2 (194)	2.798 ± 0.952				a
	3 (175)	2.851 ± 0.931				a
	4 (236)	3.524 ± 0.755				b
Social anxiety	1 (208)	3.088 ± 1.114	6.533	< 0.001	0.024	a
	2 (194)	3.230 ± 1.084				a
	3 (175)	3.224 ± 1.119				a
	4 (236)	2.823 ± 1.076				b
Peer relationships	1 (208)	2.869 ± 1.120	5.761	0.001	0.021	a
	2 (194)	2.790 ± 1.029				a
	3 (175)	2.689 ± 1.049				a
	4 (236)	3.105 ± 1.083				b

*η^2^* (eta squared) reflects the proportion of variance explained by between-group differences; 0.01 indicates a small effect, 0.06 a moderate effect, and 0.14 a large effect (Cohen, 1988). In this table, η^2^ for interpersonal skills is 0.111 (moderate effect), η^2^ for physical exercise is 0.048 (close to moderate), and *η^2^* for social anxiety and peer relationships are 0.024 and 0.021, respectively (small effects). *Post-hoc* comparisons were corrected for multiple comparisons using the *Tukey HSD* (Honest Significant Differences) test. Within the same variable, different letters (e.g., a and b) indicate that the difference in means between two groups is statistically significant (*P*< 0.05); identical letters (e.g., a and a) indicate that the difference is not significant. Taking “physical exercise” as an example, the letter for the senior class is “b,” while the letters for the freshman, sophomore, and junior classes are all “a.” This indicates that the amount of physical exercise among senior students is significantly higher than that of the other three grades, while the differences between any two of the freshman, sophomore, and junior classes are not statistically significant. The meanings of the letters for other variables (interpersonal skills, social anxiety, and peer relationships) follow the same logic.

#### 4.2.4 Pearson correlation analysis

Pearson correlation analysis was performed on data related to physical exercise, interpersonal skills among college students, social anxiety, and peer relationships (Table 5). The results showed that physical exercise was significantly positively correlated with interpersonal skills and peer relationships, and significantly negatively correlated with social anxiety. Social anxiety was significantly negatively correlated with peer relationships, and peer relationships were significantly positively correlated with interpersonal skills.

**TABLE 5:** Correlation analysis between physical exercise, interpersonal skills, social anxiety, and peer relationships.

Variables	Physical exercise	Interpersonal skills	Social anxiety	Peer relationships
Physical exercise	1	1	1	1
Interpersonal skills	0.551**			
Social anxiety	−0.571**	−0.402**		
Peer relationships	0.538**	0.387**	−0.439**	

Pearson correlation coefficient. **p<0.01.

### 4.3 Mediating effect test

As shown in Table 6, Model 6 from the SPSS PROCESS macro developed by Hayes was used to test for chained mediation. The results indicate that in the social anxiety (M1) model, the standardized regression coefficient for physical exercise on social anxiety was (β = −0.518 *p*< 0.001), suggesting that for every one-standard-deviation increase in physical exercise, social anxiety is lower by 0.518 standard deviations. In the peer relationships (M2) model, physical exercise had a significant positive predictive effect on peer relationships (β = 0.408, *p*< 0.001), while social anxiety had a significant negative predictive effect (β = −0.188, *p*< 0.001). Physical exercise and social anxiety together explained 31.7% of the variance in peer relationships (*R*^2^ = 0.317). In the interpersonal skills (Y) model, both physical exercise (β = 0.376, *p*< 0.001) and peer relationships (β = 0.102, *p*< 0.05) significantly and positively predicted interpersonal skills, while social anxiety significantly and negatively predicted interpersonal skills (β = −0.095, *p*< 0.05). Together, these three variables explained 36.4% of the variance in interpersonal skills (*R*^2^ = 0.364). After including both social anxiety and peer relationships in the PROCESS model, the direct effect of physical exercise on college students’ interpersonal skills was still significant (β = 0.376, *p*< 0.001).

**TABLE 6:** Regression analysis of the relationship of variables in the model.

Equation of regression		Overall fit index				Significance of regression coefficient		
Result variable	Variable of prediction	*R*	*R* ^2^	*F*	*Model p*	β	*t*	*p*
Social anxiety	Physical exercise	0.580	0.337	137.069	<0.001	−0.518***	−16.087	<0.001***
	Gender					0.115**	3.605	0.001**
	Grade					−0.009	−0.333	0.738
Peer relationships	Physical exercise	0.563	0.317	94.009	<0.001	0.408***	10.860	<0.001***
	Social anxiety					−0.188***	−5.282	<0.001***
	Gender					−0.051	−1.584	0.113
	Grade					–0.004	–0.163	0.870
Interpersonal skills	Physical exercise	0.603	0.364	92.512	<0.001	0.376***	9.688	<0.001***
	Social anxiety					−0.095**	−2.714	0.006**
	Peer relationships					0.102**	3.026	0.002**
	Gender					−0.085	−2.688	0.007
	Grade					0.190***	6.722	<0.001***
Interpersonal skills	Physical exercise	0.590	0.348	144.207	< 0.001	0.477***	14.956	<0.001***
	Gender					−0.103**	−3.269	0.001**
	Grade					0.191***	6.669	<0.001***

*p< 0.05, **p< 0.01, ***p< 0.001.

### 4.4 Bootstrap mediation analysis results

This study focuses on the association of physical exercise on college students’ interpersonal skills. By introducing the variables of social anxiety and peer relationships, a chain mediation model linking physical exercise and interpersonal skills among college students was constructed and validated. Both unstandardized and completely standardized effects were reported to facilitate interpretation and comparability. The results indicate (Table 7): The total effect of physical exercise on interpersonal skills is 0.0146, with a direct effect of 0.0115 and a total indirect effect of 0.0031. The mediating effects of social anxiety, peer relationships, and their interaction all reached statistical significance. Although all indirect effects were statistically significant (as indicated by 95% CIs excluding zero), the magnitudes of these effects were small to very small. The results confirm the validity of the chained mediation model, with all four hypotheses validated.

**TABLE 7:** Results of bootstrap mediated effects analysis.

Effect	Impact pathway	Effect value	Standardized effect size	BOOT SE	LLCL	ULCL	Proportion
Total effect		0.0146	0.4777	0.0010	0.0127	0.0166	100 %
Direct effect	Direct path	0.0115	0.3763	0.0012	0.0092	0.0139	78.76%
Total indirect effect		0.0031	0.1013	0.0008	0.0016	0.0047	21.24%
Indirect effect	Ind1	0.0015	0.0493	0.0006	0.0003	0.0028	10.27%
	Ind2	0.0013	0.0420	0.0005	0.0004	0.0022	8.9%
	Ind3	0.0003	0.0100	0.0001	0.0001	0.0006	2.1%

From the model, it can be seen that physical exercise is associated with interpersonal skills, and social anxiety and peer relationships play an indirect mediating role (with three paths in total). The total indirect effect value is 0.0031, and the Bootstrap 95% confidence interval does not include 0 (LLCL = 0.0016, ULCL = 0.0047), accounting for 21.24% of the total effect. Among these, the first mediating effect path: physical exercise → social anxiety → interpersonal skills (Path 1), with an indirect effect value of 0.0015, accounting for 10.27% of the total effect; the second path: physical exercise → peer relationships → interpersonal skills (Path 2), with an indirect effect value of 0.0013, accounting for 8.9% of the total effect; The third chained mediating effect path: physical exercise → social anxiety → peer relationships → interpersonal skills (Path 3) has an indirect effect value of 0.0003, accounting for 2.1% of the total effect, indicating that research hypothesis H4 holds.

**FIGURE 2:**
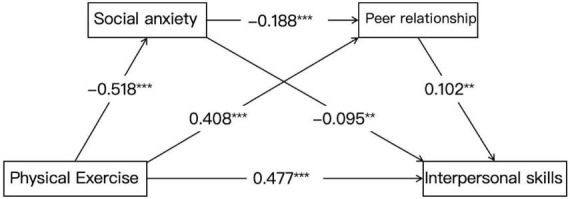
Pathways through which physical exercise affects interpersonal skills. *** Indicates a significant difference. ** Indicates a generally significant difference.

Based on the above research results, the chained mediating model is shown in Figure 2.

## 5 Discussion

This study explores the chain-mediated role of social anxiety and peer relationships in the process by which physical exercise is associated with college students’ interpersonal skills, representing a proactive exploration of how physical exercise relates to such skills. From a theoretical perspective, this study not only enriches the understanding of the mechanisms influencing interpersonal skills—particularly by conducting an in-depth analysis of the roles of social anxiety and peer relationships within these mechanisms—but also deepens our understanding of the role of physical exercise in supporting college students’ social adaptation and higher quality of their interpersonal relationships; From a practical perspective, the study provides specific pathways and theoretical support for college students to enhance their interpersonal skills by participating in physical exercise, effectively managing social anxiety, and actively building harmonious peer relationships, thereby offering strong practical guidance.

### 5.1 Physical exercise is significantly and positively associated with college students’ interpersonal skills

The results of the study indicate that physical exercise is related to interpersonal skills, and that physical exercise can significantly and positively associated with interpersonal skills. Therefore, higher levels of physical exercise demonstrate better interpersonal skills (Li et al., 2023), which is consistent with the views of previous studies. Physical exercise is not merely a form of physical exercise but also a form of mental training. Through this process, college students may indirectly be associated with better interpersonal skills (Chen, 2024). A study found that the greater the amount of physical exercise and the more frequent the exercise, the broader the interpersonal communication space (Sun and Zhang, 2020). Yan Jun’s research indicates that extracurricular physical exercise can significantly and positively predict college students’ interpersonal skills (Yan et al., 2020). Hartup argues that college students express their sincerity toward others through physical exercise during sports exercise, thereby bridging the gap between themselves and others. This is indirectly associated with higher interpersonal motivation (Hartup and Peer, 0000). During sports exercise, college students are associated with cooperation and communication with others, improve communication skills, and enhance teamwork abilities through participation in team sports or group activities. These skills play a crucial role in interpersonal interactions. Therefore, participation in physical exercise is clearly associated with better interpersonal skills among college students.

### 5.2 The mediating effect of social anxiety between physical exercise and college students’ interpersonal skills

This study found that social anxiety has a significant mediating effect between physical exercise and college students’ interpersonal skills. Specifically, physical exercise is significantly associated with lower levels of social anxiety, and lower levels of social anxiety are further associated with better interpersonal skills. This result is consistent with previous research (Wu et al., 2025). From a psychological mechanism perspective, physical exercise is associated with lower social anxiety, and this association may be explained by higher sports self-efficacy and less emotional expression inhibition (Song and Ding, 2024). Lower social anxiety is also associated with a more positive social environment, which in turn is associated with a greater willingness to participate in social activities and actively establish connections with others, and thus with better interpersonal skills (Xiang et al., 2024). Lower levels of social anxiety are associated with better college students’ interpersonal skills. Individuals with higher levels of social anxiety often exhibit more avoidance behaviors and negative emotions in social settings, which hinder their effective interaction with others (Shang et al., 2023). In summary, social anxiety plays an important mediating role in the association between physical exercise and college students’ interpersonal skills. Physical exercise is associated with lower social anxiety, which is in turn associated with a more positive psychological state and social environment, and ultimately with better interpersonal skills. This finding provides a theoretical basis for universities to implement physical exercise programs to support college students’ mental health and interpersonal skills.

### 5.3 The mediating effect of peer relationships between physical exercise and college students’ interpersonal skills

This study found that peer relationships play a significant mediating role between physical exercise and college students’ interpersonal skills. This result indicates that physical exercise is associated with better peer relationships, which in turn are associated with better interpersonal skills. This conclusion is supported by multiple studies. First, physical exercise is associated with more abundant social settings and opportunities, which are associated with the development of positive peer relationships (Li and Xie, 2019). Sports activities are typically conducted in team settings, requiring cooperation, trust, and interaction among participants, which are associated with stronger bonds and emotional connections among peers (Zhou et al., 2025). Such positive peer relationships are associated with greater social support and a sense of belonging, which in turn are associated with better interpersonal skills. Second, better peer relationships are associated with greater confidence and relaxation in social settings, which in turn are associated with more effective connections and interactions. For example, Chen and Yu (2020) research indicates that peer relationships mediate the relationship between physical exercise and college students’ subjective wellbeing, and this mediating effect is also reflected in the association with interpersonal skills. Additionally, better peer relationships are associated with lower social anxiety, which is further associated with better interpersonal skills. In summary, peer relationships play an important mediating role in the association between physical exercise and college students’ interpersonal skills. Physical exercise is associated with better peer relationships, which in turn are associated with a more positive social environment and better interpersonal skills. Beyond overall exercise volume, the specific modality and social context of physical activity may be particularly relevant for peer relationships and interpersonal skills. Team-based sports, such as basketball, soccer, or volleyball, inherently involve cooperation, communication, and shared goal-directed behaviors, which may foster peer bonding and mutual trust more effectively than individual forms of exercise such as running or swimming. Conversely, individual exercise might primarily enhance self-efficacy, emotional regulation, or stress relief, with indirect benefits for interpersonal skills that operate through improved personal wellbeing rather than direct social interaction. Nevertheless, some individual sports (e.g., martial arts, swimming) can also be practiced in group settings, and recreational physical activities often contain mixed social elements, making the distinction between “team” and “individual” less binary in practice. While our study employed a global measure of exercise volume and cannot directly compare these modalities, this distinction offers a promising avenue for future research to explore how different forms and contexts of physical exercise distinctly shape peer dynamics and interpersonal competence.

### 5.4 The chain-mediated effect of social anxiety and peer relationships on the relationship between physical exercise and college students’ interpersonal skills

When exploring the association between physical exercise and interpersonal skills, although this study identified a statistically significant chain mediation pathway through which physical exercise is associated with interpersonal skills via social anxiety and peer relationships, the absolute magnitude of the indirect effects was small, suggesting that the practical contribution of this mechanism may be limited. This indicates that physical exercise is not only directly associated with interpersonal skills but also indirectly associated through lower social anxiety and better peer relationships, highlighting the synergistic role of multidimensional psychological mechanisms. This validates existing research and reveals the complex mechanisms through which physical exercise is associated with college students’ interpersonal skills (Zhao et al., 2025). Specifically, physical exercise is associated with lower social anxiety, better peer relationships, and ultimately higher interpersonal skills. This chain-mediated process not only reflects the positive association of physical exercise with mental health but also emphasizes the important mediating role of social anxiety and peer relationships in interpersonal skills. These findings should be interpreted not only in terms of statistical significance but also in light of the magnitude of the effects. The fact that the serial mediation pathway accounted for only 2.1% of the total effect, and the total indirect effects (all three pathways combined) accounted for 21.2%, suggests that the mediating mechanisms involving social anxiety and peer relationships—though statistically significant—represent a relatively small portion of the overall association between physical exercise and interpersonal skills. This implies that the direct association between physical exercise and interpersonal skills is the dominant pathway, and that the indirect pathways, while meaningful, are unlikely to be the primary mechanisms underlying this relationship. From a practical standpoint, interventions aimed at enhancing college students’ interpersonal skills through physical exercise should not rely solely on reducing social anxiety or improving peer relationships as indirect routes; rather, the direct benefits of physical activity—such as improved mood, increased self-efficacy, and enhanced cognitive functioning—may play an equally or more important role. The small effect sizes also suggest that other unmeasured mediators, such as psychological resilience, self-efficacy, social support, and sense of belonging, may contribute to the association and warrant investigation in future research. Furthermore, it is important to acknowledge that although we proposed a directional sequence from social anxiety to peer relationships based on theoretical frameworks—which posit that internal emotional difficulties precede interpersonal difficulties —the relationship between these two constructs may be bidirectional in practice. Prior research suggests that poor peer relationships, such as peer rejection or low peer acceptance, can in turn heighten social anxiety over time, potentially creating a mutually reinforcing cycle between the two. This bidirectional perspective is well supported by the transactional model of development (Sameroff, 2009), which emphasizes the reciprocal interplay between individual characteristics and environmental experiences during social development. Given the cross-sectional nature of our data, we cannot empirically determine the direction of these relationships or rule out alternative pathways—for instance, physical exercise → peer relationships → social anxiety → interpersonal skills—nor can we exclude the possibility that these variables operate in a reciprocal, feedback-loop manner. Future research employing multi-wave longitudinal designs or cross-lagged panel models is needed to disentangle the dynamic interplay among these constructs over time. In summary, the chain-mediated effect results of this study provide a new perspective on understanding how physical exercise is associated with college students’ interpersonal skills through psychological and social factors. Future research could further explore other potential mediating variables, such as psychological resilience and self-efficacy, to more comprehensively reveal the positive associations of physical exercise with college students’ psychological and social adaptation.

## 6 Conclusion

First, there is a significant correlation between physical exercise, social anxiety, peer relationships, and interpersonal skills. Second, physical exercise can significantly and positively associated with college students’ interpersonal skills and is an important correlated factor related to college students’ social skills. Third, social anxiety and peer relationships mediate the association between physical exercise and interpersonal skills, with statistically significant but small effect sizes. The serial mediation pathway accounted for only 2.1% of the total effect; thus, in this sample, this indirect route, while statistically meaningful, accounts for a relatively small portion of the total association. The practical implications of these findings should therefore be interpreted with caution, and future research is needed to identify other potential mediators that are associated with a larger proportion of the association.

## 7 Limitations of the study and future directions

This study examines the underlying mechanisms linking physical exercise and interpersonal skills, providing a theoretical foundation for research aimed at enhancing college students’ social skills. Although the research data fits the model well, several limitations remain: ①This study employs a cross-sectional design, which does not allow for a thorough examination of causal relationships or temporal ordering among variables. Therefore, all reported associations should be interpreted as correlational, not causal. Of particular relevance to our serial mediation model, although we provided theoretical justification for the sequence of physical exercise → social anxiety → peer relationships → interpersonal skills, we cannot empirically rule out alternative directional models or bidirectional relationships (e.g., between social anxiety and peer relationships). Future research could adopt longitudinal tracking designs with at least three waves of data collection, combined with cross-lagged panel models or randomized controlled trials, to more reliably test the direction of causality and the dynamic interplay among these factors over time. ② This study relies solely on a chain mediation model to explain the relationships among the four variables. Future research could further consider alternative models to explore the relationships between these factors and academic engagement. ③ The representativeness of the sample is limited, which warrants caution when generalizing our findings to broader student populations. First, although we sampled from four provinces (Sichuan, Chongqing, Henan, and Liaoning) that span southwest, central, and northeast China, these regions do not cover the full diversity of China’s higher education landscape, particularly institutions in eastern coastal areas, northwestern provinces, or specialized vocational colleges. Cultural norms, educational policies, and physical activity environments may vary considerably across regions, potentially influencing the strength or pattern of the associations observed in our study. Second, the gender distribution in our sample (60.1% male, 39.9% female) is somewhat imbalanced compared to the overall undergraduate population in China, which has approached near-parity in recent years (approximately 51% female). Given that our data showed significant gender differences in all core variables (Table 3), this imbalance may have affected the estimated mediation effects, particularly if the relationships among physical exercise, social anxiety, peer relationships, and interpersonal skills differ systematically between male and female students. Third, our sample was drawn exclusively from 4-year undergraduate programs and did not include junior college or vocational college students, who may face different developmental challenges and physical activity contexts. Therefore, while our multi-province design enhances geographical diversity to some extent, the generalizability of our conclusions to other institutional types, geographic regions, or more gender-balanced populations should be interpreted with due caution. Future research should expand the sampling scope to include a wider range of institutions (e.g., vocational colleges, institutions in eastern coastal and western regions), deliberately recruit a more balanced gender ratio, and test whether the proposed mediation model is invariant across different subsamples through multi-group analysis. ④ Measurement errors exist but have not been fully controlled. Although the reliability (Cronbach’s α) of each scale was above 0.89, self-report measures inherently cannot avoid random and systematic errors. Furthermore, as the Physical Activity and Exercise Scale (PARS-3) is a composite indicator, its internal consistency reliability is not the most appropriate measure of quality. Future research may consider using latent variable structural equation modeling (SEM) to correct for measurement errors and obtain more precise path coefficient estimates. ⑤ The effect sizes are small, and their practical implications should be interpreted with caution. In this study, the indirect effects of chain mediation (after standardization) accounted for only 2.1–10.3% of the total effect. Although statistically significant, the effect sizes are small. Future research should test the replicability of these small effects in larger samples and explore other mediation pathways that may be stronger. In summary, future research should aim to consolidate and expand upon the preliminary findings of this study by employing longitudinal designs, multi-method measurements, more representative samples, and more advanced statistical modeling techniques (such as latent variable SEM and multi-group analysis).⑥ Limitations of the statistical methods. This study used the PROCESS macro to conduct a chained mediation analysis; this method is based on observed variables and does not account for the impact of measurement error on path estimates. Furthermore, the study assumed that the relationships in the model remain consistent across different genders or grade levels, and did not test the model’s cross-group stability. Future research could employ latent variable structural equation modeling (SEM, e.g., using Mplus software) to correct for measurement error by specifying latent variables, thereby obtaining more precise estimates of path coefficients. Additionally, multi-group analysis could be conducted to examine whether the mechanisms through which physical exercise influences interpersonal skills differ by gender or grade level. This would help clarify the model’s boundary conditions, refine the conclusions, and enhance the theoretical contributions of the study. ⑦In addition, despite the Harman single-factor test suggesting that severe common method bias was not a major concern, the possibility of common method bias cannot be completely ruled out because all data were collected via self-report questionnaires. Self-report measures are inherently susceptible to social desirability bias, recall inaccuracy, and common rater effects, which may have influenced the observed associations among variables. While we implemented procedural remedies such as temporal separation of measurements and attention-check questions to mitigate these risks, we cannot empirically quantify or eliminate the impact of such biases. Future research could strengthen causal and methodological rigor by incorporating multiple data sources, such as peer nominations or teacher evaluations for interpersonal skills and peer relationships, as well as objective physical activity monitors for exercise assessment. Multi-informant and multi-method approaches would help reduce shared method variance and provide a more robust test of the proposed mediation model.⑧The assessment of physical exercise was limited to overall volume (intensity, duration, and frequency). We did not collect detailed information on exercise type (e.g., team-based vs. individual sports), the duration of regular participation (e.g., months or years of consistent involvement), or the organizational setting (e.g., formal physical education classes, student sports clubs, or informal leisure activities). This limits our ability to examine the nuanced effects of different exercise modalities on peer relationships and interpersonal skills, and it also prevents us from determining whether the observed associations are driven primarily by the social interaction inherent in certain sports rather than by the physiological effects of exercise *per se*. Future research should incorporate more detailed measures of exercise modality, social context, and participation history to provide a more comprehensive understanding of how different forms of physical activity relate to college students’ social development. ⑨ We also acknowledge that several other potential confounding variables were not assessed or controlled for in this study. Although we included gender and academic year as covariates—consistent with prior research indicating their relevance to physical activity and interpersonal functioning—other individual and environmental factors may influence the observed associations. For instance, personality traits such as extraversion and neuroticism have been shown to correlate with both social anxiety and interpersonal competence, and self-esteem has been identified as a key psychological resource that shapes social interactions and peer relationships. Socioeconomic status, sleep quality, and academic stress may also affect students’ emotional wellbeing and social availability, thereby potentially confounding the relationships examined in our model. The absence of these variables in our study means that we cannot rule out the possibility that the observed mediation effects are partially attributable to unmeasured third variables. Future research could incorporate a broader set of covariates—including personality inventories, self-esteem measures, and indicators of stress and lifestyle—to more rigorously isolate the unique contribution of physical exercise to interpersonal skills and to enhance the explanatory power of the mediation model. ➉ Furthermore, although the indirect effects reached statistical significance, the effect sizes were relatively small, particularly for the serial mediation pathway (Ind3), which accounted for only 2.1% of the total effect. This raises the question of whether the proposed sequential mechanism, while theoretically plausible, is practically meaningful as a primary explanatory model. The small effect sizes may reflect the possibility that other unmeasured mediators—such as psychological resilience, general self-efficacy, perceived social support, and sense of belonging—play more substantial roles in linking physical exercise to interpersonal skills. We therefore encourage future research to incorporate these variables into more comprehensive models, ideally using longitudinal designs and larger samples to detect small effects with greater stability and to examine whether multiple mediators operate in parallel or in sequence. Such efforts would help clarify the relative contribution of different mechanisms and inform more targeted interventions for improving college students’ interpersonal competence.

## Data Availability

The original contributions presented in the study are included in the article/Supplementary material, further inquiries can be directed to the corresponding author.

## Footnotes

1
www.wjx.cn
